# Letter to the Editor – Update from Ukraine: Project Results in Oncology Telerehabilitation Approved at the National Cancer Institute and Showcased at the 4th National PM&R Congress

**DOI:** 10.5195/ijt.2024.6686

**Published:** 2025-01-15

**Authors:** Kyrylo S. Malakhov

**Affiliations:** Microprocessor Technology Lab, V.M. Glushkov Institute of Cybernetics of the National Academy of Sciences of Ukraine

**Keywords:** Hybrid cloud-based platform, Oncology Telerehabilitation, Breast cancer, Mathematical modeling, Artificial Intelligence, eHealth, Ukraine

## Abstract

Telerehabilitation has emerged as a vital component of oncology care, particularly under challenging conditions where access to healthcare services may be restricted. This Letter to the Editor provides an update from Ukraine on a pioneering hybrid cloud-based platform designed for the patient-centered telerehabilitation of oncology patients. We summarize the project's main outcomes, including the development of advanced AI-driven models, comprehensive methodological guidelines, and specialized software services. Notably, the platform underwent clinical approbation at the Nonprofit Organization “National Cancer Institute,” where its capacity for continuous patient monitoring, real-time data integration, and personalized rehabilitation interventions was demonstrated. Furthermore, the efficacy and innovation of this platform were showcased at the 4th National Congress of Physical and Rehabilitation Medicine in Ukraine, highlighting its potential to optimize resource allocation and improve patient outcomes. By merging mathematical and AI-based methods, the project underscores the importance of interdisciplinary collaboration in developing robust telerehabilitation ecosystems. We hope that this update contributes to the dialogue on expanding and refining telerehabilitation solutions globally, particularly in the realm of oncology care.

**January 1, 2025**

**Dear Ellen R. Cohn (PhD, CCC-SLP, ASHA-F), and Jana Cason (DHSc, OTR/L, FAOTA):**

I extend warm greetings from Ukraine on behalf of our research team and wish to share notable advancements in oncology telerehabilitation that align closely with the mission of the *International Journal of Telerehabilitation*. In this Letter to the Editor, I highlight the development and implementation of a hybrid cloud-based platform designed for patient-centered rehabilitation of oncology patients. This platform harnesses both mathematical modeling and artificial intelligence and has successfully undergone approbation at the Nonprofit Organization “National Cancer Institute” (NCI) (NO NCI, 2024b). Its outcomes were further showcased at the 4th National Congress of Physical and Rehabilitation Medicine (Congress) (*The 4th National Congress of Physical and Rehabilitation Medicine*, 2024). I believe these insights can enrich the broader discourse on telerehabilitation's role in improving both access to, and the quality of, oncology care.

## Final Results of the Project “Development of the Hybrid Cloud-Based Platform for Patient-Centered Telerehabilitation of Oncology Patients with Mathematical-Related Modeling”

The principal objective of our project (Malakhov, 2024b) was to create a hybrid cloud-based platform – along with integrated information technology (IT) solutions – to support a wide range of specialists in physical medicine and rehabilitation (PM&R) within the “Telerehabilitation of Oncology Patients” sector. A distinctive feature of this effort is the combination of artificial intelligence (AI) algorithms (Palagin, Velychko, et al., 2020), and advanced mathematical methods for discrete and non-smooth optimization. This includes developing mathematical models, subgradient space transformation algorithms to manage functions with tens of thousands of variables, and global equilibrium search methods. These strategies were implemented through sequential and parallel algorithms running on a state-of-the-art (SOTA) supercomputer complex, reflecting our group's expertise in solving sophisticated optimization problems.

The primary focus in Stage 2 of the project was to fully develop a Minimum Viable Product (MVP) for a Hybrid Cloud Environment for Telerehabilitation (HCET) (Malakhov, 2024b; Malakhov & Semykopna, 2024; Palagin, Stetsyuk, et al., 2024), refine it based on mathematical modeling and clinical approbation, and provide methodological and educational support. While the first stage concentrated on defining and justifying requirements for the HCET and its components – spanning methodological, technological, software application, and mathematical aspects – Stage 2 yielded several concrete outcomes:

- *Telerehabilitation Guidelines for Patients with Breast Cancer* (Vladymyrov et al., 2024a, 2024b). Our team formulated and approved guidelines that encompass planning and managing rehabilitation processes, including telerehabilitation methods, diagnostic procedure support, and a digital collection of rehabilitation procedure videos. These guidelines were endorsed by the Department of Physical Medicine and Rehabilitation and Sports Medicine at the Shupyk National Healthcare University of Ukraine.
- *Mathematical Models of the Rehabilitation Process* (Kaverinskiy et al., 2024; Palagin, Symonov, et al., 2024; Stetsyuk, Vakulenko, et al., 2024; Vakulenko et al., 2024). We devised non-smooth and discrete optimization models to address complex rehabilitation questions, such as optimizing the number of interventions, selecting specialists under budget constraints, and adjusting target parameters of patient health. Solutions to these models underscore the synergy between rigorous mathematical frameworks and clinical practice.
- *Refinement of the HCET Architecture* (Malakhov, 2024b)*.* Architectural, structural, and functional specifications of the HCET were modified to support a reference subsystem, and a TaskFlow Optimizer module (Palagin, Stetsyuk, et al., 2024). Through an application programming interface (API), these subsystems integrate seamlessly with the platform, enabling robust data exchange and efficient workflow management.
- *Administrative and Telerehabilitation Subsystems* (Palagin, Stetsyuk, et al., 2024)*.* We completed the development of an administrative subsystem with program modules for “Medical Reports,” “Patients,” and “Telerehabilitation.” These modules successfully underwent integration and functionality testing to align with the requirements of Ukraine's eHealth electronic healthcare system.
- *Clinical Approbation and Iterative Improvement* (NO NCI, 2024a). The HCET platform – including its telerehabilitation functions – was tested in the telerehabilitation department at the NCI. Based on clinical feedback, we fine-tuned the platform's workflows, optimizing both user experience and system performance.
- *Educational Advancements*. A training course titled “Methods and Resources for Telerehabilitation of Cancer Patients by the Example of Breast Cancer” was developed and piloted at the Department of Rehabilitation Medicine, Physical Therapy and Sports Medicine at the Shupyk National Medical University. Lectures and hands-on sessions incorporated online modules to facilitate effective teaching and learning.
- *Deployment and MVP Availability*. Following improvements guided by mathematical modeling and feedback from medical institutions, the MVP of the HCET was fully deployed on the server at the Glushkov Institute of Cybernetics. Guest access is available at https://e-rehab.pp.ua/, offering a secure, user-friendly environment for oncology telerehabilitation.
- *Implementation and Dissemination*. Project results were integrated at three levels: *Scientific* – through peer-reviewed publications, conference presentations, and presentations at the Congress; *Clinical* – via direct patient care in collaboration with the NCI; *Educational* – through the newly developed training course on telerehabilitation.

All objectives outlined in the project roadmap were achieved in full. Outcomes were disseminated in a collective monograph, methodological guidelines, more than 15 articles indexed in Scopus and Web of Science, and 12 conference proceedings. Certificates of implementation were granted by multiple institutions (Malakhov, 2024e), including the NCI, the Shupyk National Healthcare University of Ukraine, the National Specialized Children's Hospital “Ohmatdyt,” and the Government Institution “Ukrainian Medical Center of Sports Medicine.”

A list of favorite research and methodological works (articles, books, conference proceedings) published from the project results (2022 – 2024/5) follows:

- Cloud-based Platform for Patient-centered Telerehabilitation of Oncology Patients with Mathematical Related Modeling (Palagin, Stetsyuk, et al., 2024).
- Telerehabilitation Guidelines for Patients with Breast Cancer (and its Ukrainian-Language Edition) (Vladymyrov et al., 2024a, 2024b).
- Theoretical Aspects of Transdisciplinarity in Telerehabilitation (Malakhov et al., 2024).
- Algorithmization and Optimization Models of Patient-Centric Rehabilitation Programs^*^ (Vakulenko et al., 2024).
- Mathematical Modeling of the Evolution of the Rehabilitation Process for Patients with Oncological Diseases (Palagin, Symonov, et al., 2024).
- Innovative Hybrid Cloud Solutions for Physical Medicine and Telerehabilitation Research (Malakhov, 2024b).
- Integrating Hybrid Cloud Solutions in Telerehabilitation (Malakhov & Semykopna, 2024).
- Challenges and Role of Ontology Engineering in Creating the Knowledge Industry: A Research-Related Design Perspective (Palagin, Petrenko, et al., 2024).
- Fundamentals of the Integrated Use of Neural Network and Ontolinguistic Paradigms: A Comprehensive Approach (Palagin, Kaverinskiy, et al., 2024).
- Systems Science: Digitalization of Transdisciplinary Research (Petrenko & Malakhov, 2024).
- Letter to the Editor: Advancements in Digital Health Technologies (Malakhov, 2024c).
- Machine Learning Analysis of Arterial Oscillograms for Depression Level Diagnosis in Cardiovascular Health (Kaverinskiy et al., 2024).
- A Distributed Classification Method for Real-time Healthcare Data Processing (Stetsyuk, Stovba, et al., 2024).
- Use of Ellipsoid Method for Finding Linear Regression Parameters with L1-Regularization (Stetsyuk & Stovba, 2025).
- Use of Ellipsoid Method and Linear Regression with L1-Regularization for Medical Data Investigation (Stetsyuk, Stovba, et al., 2024).
- Exploring Research-Related Design: A Comprehensive Information System for Knowledge Production—OntoChatGPT (Malakhov, 2024a).
- Digital Health Systems: Ontology-Based Universal Dialog Service for Hybrid E-Rehabilitation Activities Support (Palagin, Kaverinskiy, Petrenko, et al., 2023).
- Insight into the Digital Health System of Ukraine (eHealth): Trends, Definitions, Standards, and Legislative Revisions (Malakhov, 2023a).
- Letter to the Editor – Update from Ukraine: Development of the Cloud-based Platform for Patient-centered Telerehabilitation of Oncology Patients with Mathematical-related Modeling (Malakhov, 2023b)
- OntoChatGPT Information System: Ontology-Driven Structured Prompts for ChatGPT Meta-Learning (Palagin, Kaverinskiy, Litvin, et al., 2023).
- Natural Language-Driven Dialogue Systems for Support in Physical Medicine and Rehabilitation (Kaverinsky & Malakhov, 2023).
- Ontology-driven development of dialogue systems (Litvin et al., 2023).
- Developing an Ontology-Based System for Semantic Processing of Scientific Digital Libraries (Malakhov et al., 2023).
- The Method of Using Fractal Analysis for Metastatic Nodules Diagnostics on Computer Tomographic Images of Lungs (Romaniv et al., 2023).
- Algorithm Unions for Solving Discrete Optimization Problems (Sergienko et al., 2023).
- Unified Representation of the Classical Ellipsoid Method (Stetsyuk et al., 2023)
- Hospital Information Smart-System for Hybrid E-Rehabilitation (Palagin et al., 2022).
- Telehealth in Crisis Situations – The Case of Ukraine (Malakhov, 2022).

Moving forward, the methodological framework, along with the software and hardware modules, serves as a cornerstone for patient-centered telerehabilitation of oncology patients across Ukraine. Future work will concentrate on integrating the HCET as a hospital information system that connects seamlessly with the nation's eHealth central database, thereby augmenting both clinical outcomes and operational efficiency.

## Approbation of Project Results at the Nonprofit Organization “National Cancer Institute”

On November 5, 2024, experts from the Department of Physical Medicine and Rehabilitation and Sports Medicine at the Shupyk National Healthcare University of Ukraine and scientists from the Glushkov Institute of Cybernetics of the National Academy of Sciences of Ukraine convened at the NCI (NO NCI, 2024a) to present the progress of a promising telerehabilitation initiative. Titled “Development of the Cloud-Based Platform for Patient-Centered Telerehabilitation of Oncology Patients with Mathematical-Related Modeling,” the project showcased its practical potential for advancing rehabilitation services, especially in wartime conditions and amidst restricted access to healthcare.

Olena Yefimenko, General Director of the NCI, commenced the meeting by warmly welcoming participants and underscoring the immediate need for novel digital health solutions in rehabilitation medicine. She highlighted how continuous, remote oversight of patient rehabilitation – enabled by modern IT platforms – can serve both practitioners and patients, given that many individuals seeking care are geographically dispersed.

Representatives of the project team presented key aspects of this HCET, and its supporting methodological framework:

- Oleksandr Palagin (Academician of the National Academy of Sciences of Ukraine, DSc, Full Professor, Deputy Director of V.M. Glushkov Institute of Cybernetics of the National Academy of Sciences of Ukraine) offered an overview of the project's objectives, outlining its most noteworthy results, including enhancements in telerehabilitation services informed by mathematical modeling.
- Kyrylo Malakhov (MSc, Researcher, Backend developer, DevOps engineer, a member of the expert Subgroup on Technical Issues and Architecture of Telemedicine within the Interdepartmental Working Group for the Development of the Concept of Implementation of Telemedicine in Ukraine, a member of the Coordination Scientific Council of the National Academy of Sciences of Ukraine on Artificial Intelligence) reviewed the architectural and software solutions underlying HCET, emphasizing the role of its reference subsystem components – MedRehabBot, MedLocalGPT, and the HCET digital library – in delivering evidence-based, automated support for clinicians.
- Denys Symonov (PhD, junior research fellow) introduced the TaskFlow Optimizer module (Palagin, Stetsyuk, et al., 2024), a solution that integrates machine learning, AI methods, and mathematical approaches to produce data critical for planning and managing the rehabilitation process.
- Dmytro Vakulenko (DSc, PhD, Full Professor, Head of the Department of Medical Informatics, Horbachevsky Ternopil National Medical University) provided insight into the primary HCET modules and subsystems, illustrating how a patient's rehabilitation trajectory is algorithmically generated to meet individual clinical needs.
- Oleksandr Vladymyrov (MD, DSc, Full Professor, Honored Doctor of Ukraine, head of the Department of Physical Medicine and Rehabilitation and Sports Medicine at the Shupyk National Healthcare University of Ukraine) described a specialized training course for PM&R specialists titled “Methods and Resources for Telerehabilitation of Cancer Patients by the Example of Breast Cancer,” designed to strengthen workforce capacity in digital rehabilitation.
- Mykola Budnyk (DSc, Full Professor, Chief Researcher, Department of Sensor Devices, Systems and Technologies of Contactless Diagnostics, Glushkov Institute of Cybernetics of the NAS of Ukraine, Taras Shevchenko National University of Kyiv, Assoc Prof, Dept. Computer Science, Sumy State University, Ukraine) presented the newly developed Telerehabilitation Guidelines for Patients with Breast Cancer, outlining protocols for planning, managing, and evaluating telerehabilitation programs.
- Petro Stetsyuk (Project supervisor, Corresponding Member of the National Academy of Sciences of Ukraine, Doctor of Physical and Mathematical Sciences, head of the Department of Nonsmooth Optimization Methods No. 120) concluded with an in-depth discussion of mathematical models used to optimize the cost, staffing, and target outcomes of patient rehabilitation programs. He addressed various formulations in non-smooth and discrete optimization, illustrating how collaborative clinical-mathematical research can refine resource allocation and rehabilitation strategies.

The meeting attracted more than 60 participants, including approximately 40 physical therapists and interns from the Shupyk National Healthcare University of Ukraine. A guided tour of the Department of Physical Medicine and Rehabilitation at the NCI followed, introducing attendees to the department's capabilities and operational considerations during wartime.

An active discussion concluded the event, during which stakeholders explored issues pertinent to physical and rehabilitation medicine and considered avenues for collaborative research and project expansion. Plans for subsequent meetings and joint initiatives further underscored the commitment of all parties involved to advance the quality and accessibility of oncology telerehabilitation services.

A full photo and video report from this event is available via an iCloud link (Malakhov, 2024d), providing additional insights into the presentations, discussions, and facility tour.

## Presentation and Validation at the 4th National Congress of Physical and Rehabilitation Medicine

The project team also showcased their most recent advancements at the Congress, a key event for clinicians and researchers in the PM&R field in Ukraine. Held on September 27–29, 2024, the Congress served as a platform for professionals from diverse specialties to share SOTA approaches, research outcomes, and innovative solutions.

Within the framework of the Congress, the project team organized a specialized session – Session 9: “INFORMATION SYSTEMS IN REHABILITATION” – moderated by Oleksandr Palagin. This session underscored how integrating information systems and novel technologies can enhance patient outcomes in rehabilitation. Several keynote presentations highlighted the core components and impact of the HCET:

- *Optimization Tasks for Patient-Centered Rehabilitation Programs*.
  Speaker: Petro Stetsyuk.
  Focused on mathematical models and non-smooth optimization problems that enhance resource allocation and customize the rehabilitation program to the patient's needs and budget constraints.
- *Modern Review of Personalized Telerehabilitation Ecosystem in Ukraine: Prospects and Challenges*.
  Speaker: Dmytro Vakulenko.
  Explored the broader telerehabilitation landscape, discussing key enabling technologies, lessons learned, and future directions for personalized rehabilitation services.
- *Cloud Platform for Patient-Centered Telerehabilitation of Cancer Patients Based on Mathematical Modelling.*
  Speaker: Oleksandr Palagin.
  Detailed the goals, architecture, and outcomes of the HCET project, illustrating how advanced modeling methods significantly enhance patient monitoring, therapy adjustments, and clinical decision-making.
- *Ethical Uses of New Technologies in Physical Medicine and Rehabilitation.*
  Speaker: Special Guest Ellen R. Cohn (pre-recorded keynote).
  Examined the ethical implications of integrating AI-based tools and remote monitoring systems into PM&R practice, emphasizing the importance of patient privacy, data security, and responsible innovation.
- *Methodological Principles of Telerehabilitation Programs for Cancer Patients.*
  Speaker: Yaroslava Zakomorna (Senior Ergotherapist, NCI).
  Addressed the clinically grounded processes essential for establishing effective, evidence-based telerehabilitation pathways, particularly for oncology patients who may face mobility and accessibility barriers.

During the session, audience members engaged in thoughtful discussion regarding the feasibility, scalability, and sustainability of telerehabilitation interventions. The project's results, encompassing evidence-based guidelines, digital infrastructure, and preliminary clinical outcomes, further solidified the value of the HCET platform in modernizing cancer rehabilitation services. By presenting these findings to a specialized PM&R audience, the project secured another layer of validation, demonstrating its potential for broader application and integration in Ukraine's healthcare ecosystem.

A live recording of Session 9: “INFORMATION SYSTEMS IN REHABILITATION” is available via YouTube (Palagin, 2024). The full set of session recordings from the Congress can also be accessed on the official Congress website, at (*The 4th National Congress of Physical and Rehabilitation Medicine*, 2024).

A full photo report, and the Congress program are available via iCloud link (*The 4th National Congress of Physical and Rehabilitation Medicine Photo Report*, 2024).

**Sincerely,**

**Kyrylo Malakhov**

**On behalf of the Institute's research team**

**Glushkov Institute of Cybernetics, National Academy of Sciences of Ukraine**
